# Clinicopathological characteristics of patients with paraproteinemia and renal damage

**DOI:** 10.1186/s40001-021-00538-2

**Published:** 2021-07-03

**Authors:** Xuanli Tang, Feng Wan, Jin Yu, Xiaohong Li, Ruchun Yang, Bin Zhu

**Affiliations:** Department of Nephrology, Key Laboratory of Kidney Disease Prevention and Control Technology, Hangzhou TCM Hospital Affiliated to Zhejiang Chinese Medical University, Hangzhou, 310007 China

**Keywords:** Paraproteinemia, Monoclonal gammopathy of renal significance, Multiple myeloma, Monoclonal gammopathy of undetermined significance, Renal monotypic immunoglobulin

## Abstract

**Background:**

This study aimed to analyze the clinicopathological characteristics of patients with paraproteinemia and renal damage.

**Methods:**

Ninety-six patients from 2014 to 2018 with paraproteinemia and renal damage were enrolled and the clinical data, renal pathology, treatment and prognosis data were collected.

**Results:**

A total of 96 patients (54 male and 42 female), accounting for 2.7% of all renal biopsies, were enrolled in this study. Among them, 42 were monoclonal gammopathy of renal significance (MGRS), 21 were renal monotypic immunoglobulin alone (renal monoIg), and 19 were monoclonal gammopathy of undetermined significance (MGUS). Individuals with multiple myeloma (MM) accounted for the fewest number of patients (*n*  =  14). In the MGRS group, the main diseases were amyloidosis (*n*  =  25) and cryoglobulinemic glomerulonephritis (*n*  =  7), while in the MM group, the main diseases were cast nephropathy (*n*  =  9) and light chain deposit disease (*n*  =  3). In the MGUS group, it was mainly IgA nephropathy (IgAN, *n*  =  10) and idiopathic membranous nephropathy (*n*  =  5); while in the renal monoIg group, most of the cases were IgAN (*n*  =  19). Chemotherapy was mainly administered to patients in the MM group, while immunosuppression therapy was mostly administered to patients in the renal monoIg group. Most patients with renal monoIg exhibited a major response, followed by the patients with MGUS and MGRS, while most of the patients with MM had a partial response but none had a major response. Approximately more than half (57.1%) of the patients with MM progressed to end-stage renal disease (ESRD), followed by MGRS (33.3%); however, the mortality rate was low in both the MGRS and MM groups. The survival analysis reviewed that serum creatinine, hemoglobin levels, and the serum *κ*/*λ* ratio were independent risk factors for ESRD in patients with MGRS.

**Conclusions:**

The clinicopathological changes in patients with MGRS were between those in patients with MM and MGUS. The treatment for MGRS and MM was more intensive, and the overall mortality rate was low. Both MGUS and renal monoIg alone exhibited slighter clinicopathological features than MGRS and MM, and the treatment was focused mostly on primary renal diseases.

## Introduction

The detection rate of monoclonal immunoglobulin (MIg) has increased due to technological advances and the widespread use of serum and renal paraprotein analysis. Accordingly, renal damage with paraproteinemia is commonly diagnosed among older patients. A paraprotein is an MIg or immunoglobulin fragment, which is detected as abnormal serum or urine light and/or heavy chains, or an abnormal light chain ratio. Paraproteins that originate from disorders of the plasma and/or B cells are known as monoclonal gammopathy of undetermined significance (MGUS). MGUS is defined as the presence of a serum monoclonal protein at a concentration of 3.0 g per deciliter or less, no monoclonal protein or only modest amounts of monoclonal light chains in the urine, the absence of CRAB features (i.e., hypercalcemia, renal insufficiency, anemia, and bone lesions) that are related to the monoclonal protein, and 10% or fewer monoclonal plasma cells in the bone marrow [1]. According to data from the Mayo Clinic in the United States, the incidence rate of MGUS can be as high as 3% in Caucasians aged over 50 years [2, 3]. These patients have no definite lymphocytic disease or do not meet the diagnostic criteria; however, the risk of progressing to lymphocytic hematopoietic malignancies is 1% per year, and the risk of progression after 25 years is 30% [4, 5]. MIg is more common in elderly patients aged over 70 years, which might be more possibly combined with immune system disorders, infections, somatic malignancies, or primary kidney diseases [1, 6, 7]. Monoclonal gammopathy of renal significance (MGRS), which refers to monoclonal immunoglobulinemia-associated kidney injury, mostly progresses to renal dysfunction (approximately 72%), but it can also develop into multiple myeloma (MM; approximately 18%). Additionally, patients with MGUS progress to more advanced disease at a rate of 1% per year, and approximately 3% of patients developed MM out of MGUS [8]. Furthermore, some patients exhibited monotypic immunoglobulin in renal tissue (renal monoIg) by light chain detection, but without MIg in serum. The cause and pathogenesis of renal monoIg is controversial [9–11]. Due to the large differences in kidney damage and prognosis, our study aimed to compare some serological and renal pathological characteristics as well as clinical treatment and prognosis of patients with paraproteinemia and renal damage, especially those with MGRS, MM, MGUS, and renal monoIg.

## Materials and methods

### Patients

**Fig. 1:**
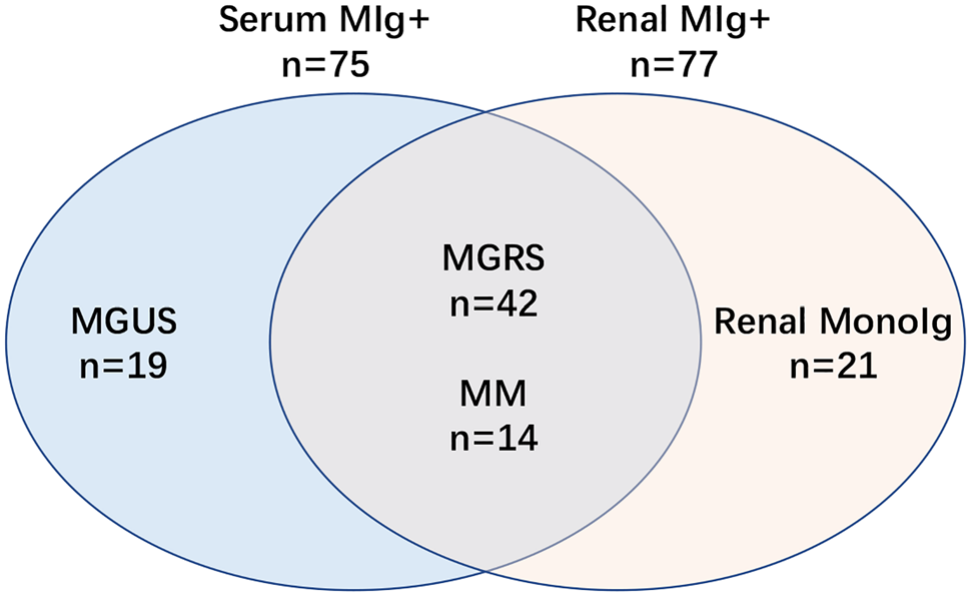
Distribution of patients with renal damage who were serum monoclonal immunoglobulin or renal monotypic immunoglobulin-positive

Of the 3502 patients who had renal biopsy in our hospital between 2014 and 2018, 96 patients were enrolled. The presence of MIgs, based on hematology findings, or monotypic immunoglobulins, based on renal biopsy findings, were detected in these patients. Patients were divided into four groups according to different diagnostic criteria. Group 1: MGRS (*n*  =  42); a clonal plasma cell or B lymphocyte proliferation causing a renal lesion in the absence of hematologic malignancy or other myeloma-defining events [12]. Group 2: MM (*n*  =  14); clonal bone marrow plasma cells  ≥  10% and the presence of one or more of myeloma-defining events; clonal plasma cells  ≥  60%; serum free light chain ratio of  ≥  100; or more than one focal lesion on magnetic resonance imaging [13]. Group 3: MGUS (*n*  =  19);  <  10% bone marrow plasma cells,  <  3 g/dl of monoclonal protein, and no myeloma-defining events [1, 13]. Group 4: the renal monoIg (*n*  =  21); the presence of monotypic immunoglobulin in renal tissue without the presence of serum MIg. The indication for kidney biopsy in monoIg group was undetermined hematuria and/or proteinuria. Patients with a history of malignancy treatment, those lost to follow-up, and those with incomplete laboratory and pathological data (*n*  =  3) were excluded. The distribution of cases is shown in Fig. 1. This study was approved by the ethical committees of Hangzhou TCM Hospital Affiliated to Zhejiang Chinese Medical University.

### Detection of serum monoclonal immunoglobulin

Serum light chain assays were conducted using an automatic protein analysis system (Siemens, Erlangen, Germany). Immunofixation electrophoresis was performed using an automated Scanning system (Hydrasys 2, Sebia, Lisses, France). All detection tests were based on the manufacturer’s instructions.

### Detection of renal monotypic immunoglobulin

Immunofluorescence (IF) of light chains was performed on paraffin-embedded tissues. The tissue was dewaxed, digested with proteinase K (Dako, Carpinteria, CA), and reacted with polyclonal rabbit anti-human *κ* and *λ* antibodies (1:40 dilution, Dako, Carpinteria, CA, USA). Light chain detection was evaluated and confirmed by two renal pathologists. Some cases of light chain detection were confirmed using immunoelectron microscopy. Sections measuring 100 nm were cut using an ultramicrotome and reacted with light chains, and then labeled with immunogold goat anti-rabbit IgG (1:40 dilution, EMS, USA). Sections were then detected under a JOEL-1400 electron microscope (JEOL-1400, JEOL Ltd., Peabody, MA, USA).

### Clinical and pathological characteristics

The following demographic and clinicopathological information was collected as followed: age, sex, renal lesion duration, hematuria, 24-h proteinuria, serum albumin, hemoglobin (Hb), serum creatinine (SCr), the estimated glomerular filtration rate (eGFR) (Modification of Diet in Renal Disease formula), serum C3 level, serum/urine *κ*/*λ* ratio and serum/urine immunofixation electrophoresis (sIFE/uIFE). Pathological features included the diagnosis, the proportion of global and segmental sclerosis, and crescents as well. Interstitial fibrosis/tubular atrophy (IFTA) and interstitial inflammation were estimated according to the proportion of cortex involved and they were divided into three grades (≤  25% as score 1, 25–50% as score 2, and  ≥  50% as score 3). Podocyte effacement was divided into three grades (≤  50% as score 1, 50–75% as score 2,  ≥  75% as score 3). Serum and urine electrophoresis, ANA, ANCA were performed on all patients and anti-PLA2R antibody was performed on patients with idiopathic membranous nephropathy (IMN). Bone marrow aspiration/biopsy was performed on patients with paraproteinemia. The final clinical diagnosis was based on laboratory tests and pathology reports.

### Treatment and outcome

Special treatments included chemotherapy, immunosuppression, and renal replacement (including long-term dialysis and renal transplantation). Chemotherapy regimens consisted mostly of bortezomib combined with dexamethasone, thalidomide combined with dexamethasone, or vincristine and adriamycin combined with dexamethasone. Immunosuppressive drugs consisted mainly of steroids, cyclophosphamide, mycophenolate mofetil, and rituximab. Renal response was defined as major response, partial response, and endpoints [death or end-stage renal disease (ESRD)]. Major response was defined as a reduction in proteinuria to protein excretion  <  0.3 g/d with stable or improvement of eGFR to  ≥  60 mL/min/1.73 m^2^; partial response was defined as  ≥  50% decrease (by at least 0.5 g) in 24-h urine protein excretion in the absence of a reduction in eGFR  ≥  25% or an increase in Scr level  ≥  0.5 mg/dL [14, 15]**.** ESRD was defined as Scr level  >  6.0 mg/dL or being dependent on kidney replacement therapy for more than 6 months [16]. Patients were followed up in the outpatient clinic or by telephone. A combination of ESRD and all-cause death was defined as the endpoint in the Kaplan–Meier analysis.

### Statistical analysis

Statistical analysis was performed with SPSS 17.0. Normal distribution data were expressed as mean  ±  standard deviation (SD). The clinical and pathological characteristics among the groups were compared by *t *test or analysis of variance (ANOVA) for continuous variables, and by nonparametric tests for discontinuous variables. Categorical variables were expressed as percentages and between-group comparisons were assessed by Chi-square test or Fisher’s exact test. Death and renal endpoints were analyzed by univariate Cox regression followed by multivariate Cox regression. Predictive models were created using clinical data and pathological data. Univariate variables included the following variables: age, gender, lesion duration, hematuria, proteinuria, albumin, Hb, Scr, eGFR, low serum C3 level, abnormal serum and urine *κ*/*λ* ratio, abnormal sIFE and uIFE, presence of renal monotypic *κ*, glomerular global and segmental sclerosis, crescents, IFTA, interstitial inflammation, and podocyte effacement. Multivariate variables included Hb, Scr, eGFR, abnormal serum and urine *κ*/*λ* ratio, and glomerular global and segmental sclerosis. The Kaplan–Meier analysis was used to compare the survival rates. Results were expressed as hazard ratio with 95% confidence intervals. All *P *values were two-sided, and statistical significance was set at *P * <  0.05.

## Results

### Demographic and clinicopathological data of patients with MGRS, MM, MGUS, and renal monoIg

**Fig. 2:**
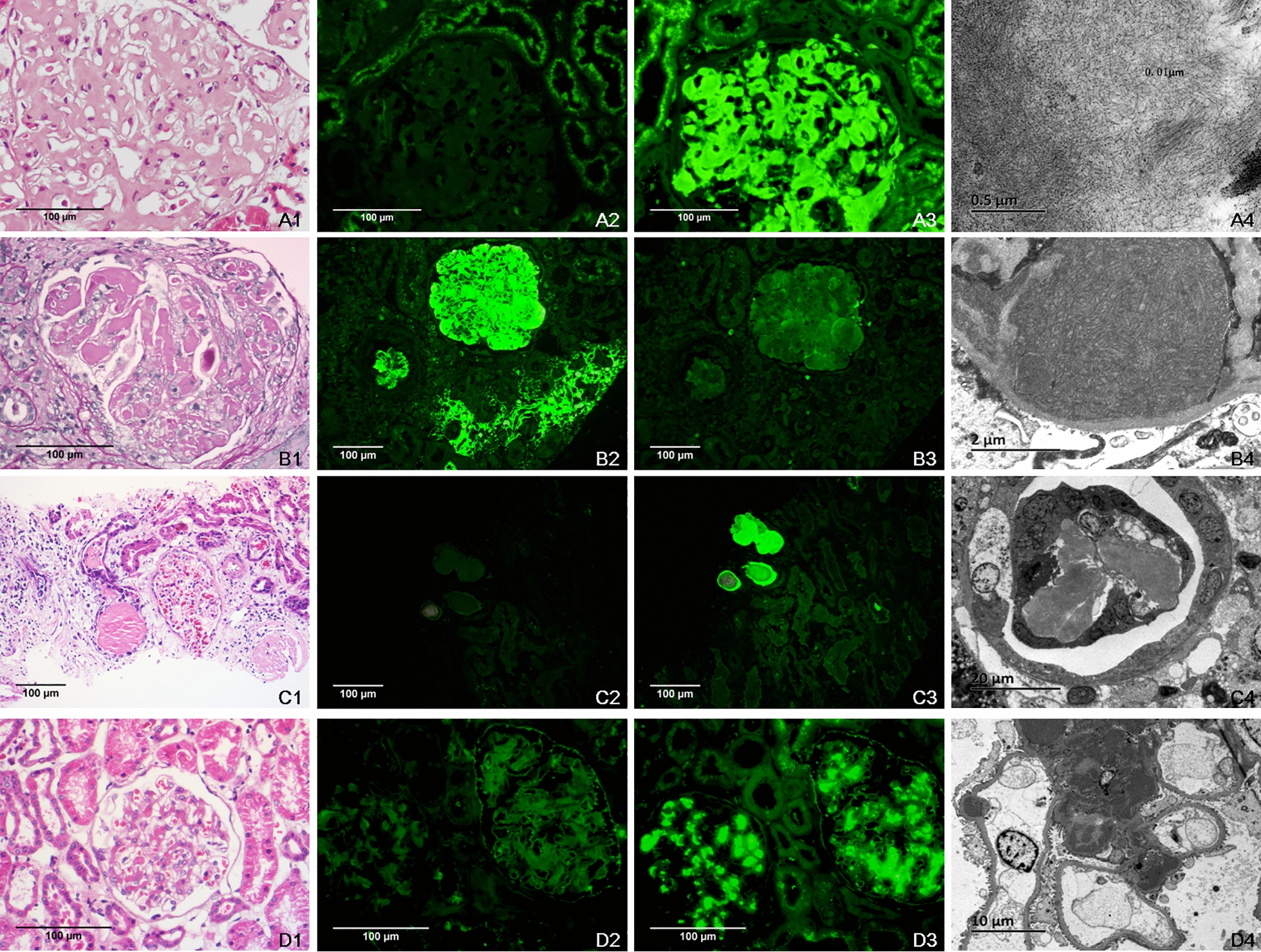
Renal pathological characteristics in some patients with serum monoclone or renal monoIg positivity. **A**–**D** Representative cases of amyloidosis, cryoglobulinemic glomerulonephritis, cast nephropathy and light chain restricted IgA nephropathy. **A**1 The glomerulus exhibited expansion of the mesangium and thickening of capillary walls by amorphous material (HE,  ×  400); **A**2 negative *κ* light chain and **A**3 positive *λ* light chain (IF,  ×  400); **A**4 8–12 nm nonbranching fibrils in the subepithelial and mesangial areas (EM,  ×  40,000); **B**1 the glomerulus exhibited cryoglobulin pseudothrombi in the glomerular capillary lumens (HE,  ×  400); **B**2 positive *κ* light chain and **B**3 negative *λ* light chain (IF,  ×  200); **B**4 large and curved fibrils seen in subendothelial deposits (EM,  ×  10,000); **C**1 a large eosinophilic and fractured cast with interstitial inflammation (HE,  ×  200); **C**2 negative *κ* light chain and **C**3 positive *λ* light chain (IF,  ×  200); **C**4 fractured cast with multinucleated giant cell reaction (EM,  ×  1000); **D**1 the glomerulus showed mesangial proliferation (HE,  ×  400); **D**2 negative *κ* light chain and **D**3 positive *λ* light chain (IF,  ×  400); **D**4 mesangial expansion with amorphous dense deposits (EM,  ×  2000)

A total of 96 patients (54 male and 42 female), were enrolled in this study, which accounted for 2.7% of all renal biopsies, performed in our hospital during the study period. The mean age of the patients was 53.3  ±  12.8 years, and the participants were divided into four groups. MGRS accounted for 43.7% (*n*  =  42) of all cases, followed by the renal monoIg and MGUS groups (21.9% and 19.8%, respectively). MM accounted for the fewest number of cases (14.6%). In the MGRS group, amyloidosis was the main disease, affecting 59.5% (*n*  =  25) of cases. In the MM group, cast nephropathy was the main disease (*n*  =  9), and three of these cases had light chain deposit disease (LCDD) combined with MM (Fig. 2). In the MGUS group, patients were mainly diagnosed with primary or secondary IgA nephropathy (IgAN) (*n*  =  8 and *n*  =  2, respectively), followed by IMN (*n*  =  5), mesangial proliferation nephritis (*n*  =  2), acute tubular injury (*n*  =  1), and malignant hypertension (*n*  =  1). In the renal monoIg group, most of the cases had IgAN (*n*  =  19). The remaining two cases were lupus nephritis (LN) and hepatitis B-related nephritis. A comparison of the clinicopathological characteristics is shown in Table 1. Among those four groups, patients with MGRS were found to exhibit lower levels of albumin and worse podocyte effacement compared to the other groups (*P * <  0.001). Additionally, the sIFE-positive detection rate was lower in the MGRS group than in the MM and MGUS groups (*P * <  0.001). Patients with MM exhibited a much lower levels of hemoglobin, yet less hematuria (*P * <  0.001 and *P * =  0.021, respectively), the highest SCr levels and the lowest eGFR than some of the other groups (*P * =  0.002 and *P * =  0.054, respectively). Additionally, patients with MM had a higher abnormal *κ*/*λ* ratio in both the serum and urine compared to the other groups (*P * =  0.024), and their interstitial fibrosis and inflammation were worse (*P * =  0.029). The MGUS group exhibited the highest levels of hemoglobin and the lowest levels of serum creatinine (*P * <  0.001 and *P * =  0.002, respectively). No renal monoclonal light chain deposits were detected. Renal monoIg patients were the youngest, and had more hematuria and less proteinuria than some of the other groups (*P * <  0.001, *P * =  0.021, and *P * =  0.026, respectively). No hematological paraproteins were detected according to the abnormal light chain ratio or IFE.

**Table 1:** Clinicopathological characteristics of patients with paraproteinemia and renal damage

	MGRS (*n* = 42)	MM (*n* = 14)	MGUS (*n* = 19)	Renal monoIg (*n* = 21)	*P *value
Male [*n* (%)]	25 (59.5)	9 (64.3)	12 (63.2)	8 (38.1)	0.292
Age (year)	57.0 ± 10.6	57.0 ± 7.0	53.2 ± 11.5	43.3 ± 15.9^c,e,f^	0.000
Renal disease duration before renal biopsy (months)	11.0 ± 11.1	6.3 ± 8.1	21.1 ± 44.1	26.8 ± 57.5	0.210
Hematuria [*n* (%)]	12 (27.9)	2 (15.4)^d^	10 (52.6)	12 (57.1)^c,e^	0.021
Proteinuria (g/24 h)	3.9 ± 2.7	3.3 ± 2.6	2.8 ± 2.6	1.8 ± 2.3^c^	0.026
Albumin (g/L)	27.7 ± 7.0^a,b,c^	36.6 ± 6.8	32.7 ± 7.4	35.3 ± 7.1	0.000
Hemoglobin (g/L)	113.6 ± 21.3	99.9 ± 16.7^a,d,e^	131.2 ± 16.3^b^	122.9 ± 19.6	0.000
SCr (μmol/L)	160.2 ± 205.8	359.1 ± 385.4^a,d,e^	102.6 ± 103.5	104.1 ± 60.2	0.002
eGFR (mL/min/1.73m^2^)	68.9 ± 39.7	45.3 ± 43.6^a,d,e^	77.7 ± 25.4	75.3 ± 34.9	0.054
Low serum C3 [*n* (%)]	15 (35.7)	3 (21.4)	1 (5.3)^b,f^	7 (33.3)	0.078
Abnornal serum *κ*/*λ* ratio [*n* (%)]	19 (45.2)	11 (78.6)^a,d^	8 (42.1)	0 (0)^c,e,f^	0.000
Abnormal urine *κ*/*λ* ratio [*n* (%)]	17 (42.5)	13 (92.9)^a,d^	5 (29.4)	0 (0)^c,e,f^	0.000
Abnormal sIFE [*n* (%)]	25 (61.0)^a,b^	13 (92.9)	19 (100)	0 (0)^c,e,f^	0.000
Abnormal uIFE [*n* (%)]	23 (76.7)	12 (85.7)	8 (53.3)	0 (0)^c,e,f^	0.002
*κ* sIFE, [*n* (%)]	9 (37.5)	7 (53.8)	11 (57.9)	0 (0)	0.371
*κ* uIFE, [*n* (%)]	5 (22.7)	6 (50.0)	4 (50.0)	0 (0)	0.183
Renal monotypic *κ* [*n* (%)]	16 (40.0)	7 (53.8)	0 (0)^b,d^	0 (0)^c,e,f^	0.000
Global glomerulosclerosis (%)	16.7 ± 18.0	17.9 ± 24.3	13.3 ± 10.1	15.1 ± 16.0	0.863
Segmental glomerulosclerosis (%)	0.8 ± 3.7	1.1 ± 1.9	1.2 ± 3.0	1.5 ± 2.9	0.223
Crescents (%)	4.1 ± 14.4	2.2 ± 3.8^e^	4.1 ± 7.6	13.3 ± 2.9	0.024
IFTA	1.29 ± 0.84	2.0 ± 1.18^a,d^	1.05 ± 0.71	1.29 ± 0.90	0.029
Interstitial inflammation	1.20 ± 0.90	2.00 ± 1.17^a,d^	1.11 ± 0.81	1.52 ± 1.03	0.000

*MGRS* monoclonal gammopathy of renal significance; *MM* multiple myeloma; *MGUS* monoclonal gammopathy of undetermined significance; *MIg* monoclonal immunoglobulin; *monoIg* monotypic immunoglobulin; *SCr* serum creatinine; *eGFR* the estimated glomerular filtration rate; *sIFE* serum immunofixation electrophoresis; *uIFE* urine immunofixation electrophoresis; *IFTA* interstitial fibrosis/tubular atrophy

^a^*P * <  0.05, MGRS vs MM group

^b^*P * <  0.05, MGRS vs MGUS group

^c^*P * <  0.05, MGRS vs renal monoIg group

^d^*P * <  0.05, MM vs MGUS group

^e^*P * <  0.05, MM vs renal monoIg group

^f^*P * <  0.05, MGUS vs renal monoIg group

### Clinicopathological features of patients with MGRS

Most patients with MGRS had amyloidosis (n  =  25), followed by cryoglobulinemic glomerulonephritis (Cryo-GN, *n*  =  7), proliferative glomerulonephritis with monoclonal IgG deposits (PGNMID, *n*  =  5), and monoclonal immunoglobulin deposition disease (MIDD, *n*  =  3). The clinicopathological data of these patients are summarized in Table 2. Patients with C3 glomerulonephritis (C3GN) and immunotactoid glomerulonephritis (ITG) were excluded due to the limited number of cases (only one case each). Patients with amyloidosis had less hematuria than those with Cryo-GN or PGNMID (*P * =  0.001), and their Hb level was the highest (*P * <  0.001). Patients with Cryo-GN had the highest level of proteinuria while those with MIDD had the highest albumin levels (*P * =  0.002 and *P * =  0.017, respectively). Patients with MIDD had the highest SCr levels and the lowest eGFR (*P * =  0.002 and *P * =  0.001, respectively), followed by those with Cryo-GN. More patients with PGNMID had low serum C3 levels than those with amyloidosis. The abnormal *κ*/*λ* ratio and IFE were similar among the groups. Patients with amyloidosis exhibited a lower proportion of serum and renal monoclonal *κ* chains than those with Cryo-GN and PGNMID (*P * =  0.031 and *P * =  0.003, respectively). Patients with MIDD exhibited worse chronic pathological features, including interstitial fibrosis and inflammation (*P * <  0.001).

**Table 2:** Clinicopathological characteristics of patients in MGRS group

	Amyloidosis (*n* = 25)	Cryo-GN (*n* = 7)	MIDD (*n* = 3)	PGNMID (*n* = 5)	*P *value
Male [*n* (%)]	17 (68.0)	4 (57.1)	2 (66.7)	2 (38.1)	0.336
Age (year)	58.0 ± 7.7	53.3 ± 16.4	69.7 ± 8.4	56.4 ± 7.5	0.209
Renal disease duration before renal biopsy (months)	10.0 ± 10.8	7.8 ± 7.0	22.0 ± 22.7	13.7 ± 10.3	0.289
Hematuria [*n* (%)]	2 (23)^a,c^	6 (85.7)	1 (33.3)	3 (60.0)	0.001
Proteinuria (g/24 h)	3.8 ± 2.3	7.0 ± 2.9^a,d,e^	2.0 ± 1.6	2.1 ± 1.5	0.002
Albumin (g/L)	25.8 ± 5.8	25.4 ± 3.9	36.6 ± 1.7^b,d^	31.1 ± 9.7	0.017
Hemoglobin (g/L)	123.4 ± 16.5^a,b,c^	91.1 ± 12.5	93.0 ± 28.2	104.6 ± 16.5	0.000
SCr (μmol/L)	134.2 ± 216.8	243.3 ± 197.3	386.3 ± 221.0^b,f^	78.6 ± 33.1^e^	0.002
eGFR (mL/min/1.73m^2^)	81.7 ± 37.6	29.0 ± 17.1^a,e^	18.4 ± 10.7 ^b,f^	80.1 ± 30.4	0.001
Low serum C3 [*n* (%)]	4 (16.0)^c^	3 (42.9)	2 (66.7)	5 (100)	0.089
Abnormal serum *κ*/*λ* ratio [*n* (%)]	13 (52.0)	3 (42.9)	2 (66.7)	0 (0)	0.269
Abnormal urine *κ*/*λ* ratio [*n* (%)]	14 (60.9)	1 (14.3)	2 (66.7)	0 (0)	0.131
Abnormal sIFE [*n* (%)]	16 (66.7)	5 (71.4)	2 (66.7)	1 (20)	0.237
Abnormal uIFE [*n* (%)]	17 (77.3)	3 (75)	2 (100)	1 (50)	0.702
*κ* (sIFE, [*n* (%)])	3 (20.0)^a^	4 (80)	1 (50)	0 (0)	0.031
*κ* (uIFE, [*n* (%)])	3 (18.8)	1 (33.3)	1 (50.0)	0 (0)	0.687
Renal monotypic *κ* [*n* (%)]	4 (16.0)^c^	5 (71.4)	2 (66.7)	4 (80)	0.003
Global glomerulosclerosis (%)	14.4 ± 16.4	17.4 ± 22.8	42.3 ± 13.7^b,d,f^	12.6 ± 13.8	0.081
Segmental glomerulosclerosis (%)	0	3.1 ± 8.3	0	2 ± 4.5	0.236
Crescents (%)	0	18.0 ± 32.2^a^	0	8.2 ± 6.8	0.319
IFTA	1.04 ± 0.69^a^	1.86 ± 0.69^e^	3.0 ± 0.0^b,d,f^	1.0 ± 0.0	0.000
Interstitial inflammation	0.79 ± 0.66^a,b^	2.14 ± 0.69	2.33 ± 0.58	1.40 ± 0.89	0.000
Podocyte effacement (%)	2.6 ± 0.6	2.6 ± 0.8	2.3 ± 0.6	2.4 ± 0.9	0.883

*MGRS *monoclonal gammopathy of renal significance; *MM* multiple myeloma; *MGUS* monoclonal gammopathy of undetermined significance; *MIg* monoclonal immunoglobulin; *monoIg* monotypic immunoglobulin; *SCr* serum creatinine; *eGFR* the estimated glomerular filtration rate; *sIFE* serum immunofixation electrophoresis; *uIFE* urine immunofixation electrophoresis; *IFTA* interstitial fibrosis/tubular atrophy

^a^*P * <  0.05, Amyloid vs Cryo group

^b^*P * <  0.05, Amyloid vs MIDD group

^c^*P * <  0.05, Amyloid vs PGNMID group

^d^*P * <  0.05, Cryo vs MIDD group

^e^*P * <  0.05, Cryo vs PGNMID group

^f^*P * <  0.05, MIDD vs PGNMID group

### Treatment and survival analysis of patients with MGRS, MM, MGUS and renal monoIg

Most of the patients in the four groups underwent special treatment, including chemotherapy and immunosuppression (Table 3). Chemotherapy was administered mostly to patients with MM, and it was administered significantly more to patients with MM than to those with MGRS (*P * <  0.001). Immunosuppression therapy was administered mostly to patients in the renal monoIg group, followed by those in the MGUS and MGRS groups, each of which were significantly different (*P * <  0.001). Long-term dialysis or renal transplantation was only applied in patients with MGRS and MM who had ESRD. The average follow-up period was 44.8  ±  16.9 months. The renal monoIg group had the highest proportion of patients with major response, followed by the MGUS and MGRS groups, each of which were significantly different (*P * <  0.001). The MM group had a higher proportion of patients with partial response, but none of the patients showed a major response. More than half of the patients with MM (57.1%) reached ESRD, followed by those with MGRS (33.3%), although both the MGRS and MM groups had a low mortality rate without significant difference (16.7% vs 21.4%, *P * >  0.05). The difference in survival time between these patients was also not significant (16.7% vs. 21.4%). Patients with MGUS demonstrated a very low rate of ESRD progression, and none of the patients with MGUS or renal monoIg died during the observational time.

**Table 3:** Treatment and survival analysis of patients with paraproteinemia and renal damage

	MGRS (*n* = 42)	MM (*n* = 14)	MGUS (*n* = 19)	Renal monoIg (*n* = 21)	*P* value
Special treatment [*n* (%)]	36 (85.7)	13 (92.9)	13 (68.4)	18 (85.7)	0.239
Chemotherapy [*n* (%)]	19 (45.2)^a,b,c^	12 (85.7)^d,e^	0 (0)	0 (0)	0.000
Immunosuppression [*n* (%)]	12 (28.6)^a,b,c^	0 (0)^d,e^	12 (63.2)	18 (85.7)	0.000
Follow-up time (months)	47.6 ± 19.2	50.9 ± 18.4	38.9 ± 14.7	40.4 ± 10.0	0.080
Major response [*n* (%)]	10 (23.8)^a,b,c^	0 (0)^d,e^	11 (57.9)^f^	18 (85.7)	0.000
Partial response [*n* (%)]	16 (38.1)	6 (42.9)	5 (26.3)	3 (14.3)	0.185
ESRD [*n* (%)]	14 (33.3)^b^	8 (57.1)^d,e^	1(5.3)	0 (0)	0.025
Baseline eGFR (mL/min)	50.0 (14.7, 89.0)	15.6 (5.8, 34.7)	22	–	0.732
Renal survival time (months)	8.8 ± 10.0	9 ± 16.6	2	–	0.507
Death [*n* (%)]	7 (16.7)^b,c^	3 (21.4)^d,e^	0 (0)	0 (0)	0.014
Survival time (months)	9.4 ± 8.4	8.0 ± 6.1	–	–	0.651

Baseline eGFR and renal survival time were of ESRD patients, while survival time was of died patients

Special treatments included chemotherapy, immunosuppression, and renal replacement (including long-term dialysis and renal transplantation). Chemotherapy regimens consisted mostly of bortezomib combined with dexamethasone (BD regimen), thalidomide combined with dexamethasone (TD regimen), or vincristine and adriamycin combined with dexamethasone (VAD regimen). Immunosuppressive drugs consisted mainly of steroids, cyclophosphamide, mycophenolate mofetil, and rituximab

*ESRD* end-stage renal disease, *IS* immunosuppression

^a^*P * <  0.05, MGRS vs MM group

^b^*P * <  0.05, MGRS vs MGUS group

^c^*P * <  0.05, MGRS vs renal monoIg group

^d^*P * <  0.05, MM vs MGUS group

^e^P  <  0.05, MM vs renal monoIg group

^f^P  <  0.05, MGUS vs renal monoIg group

**Fig. 3:**
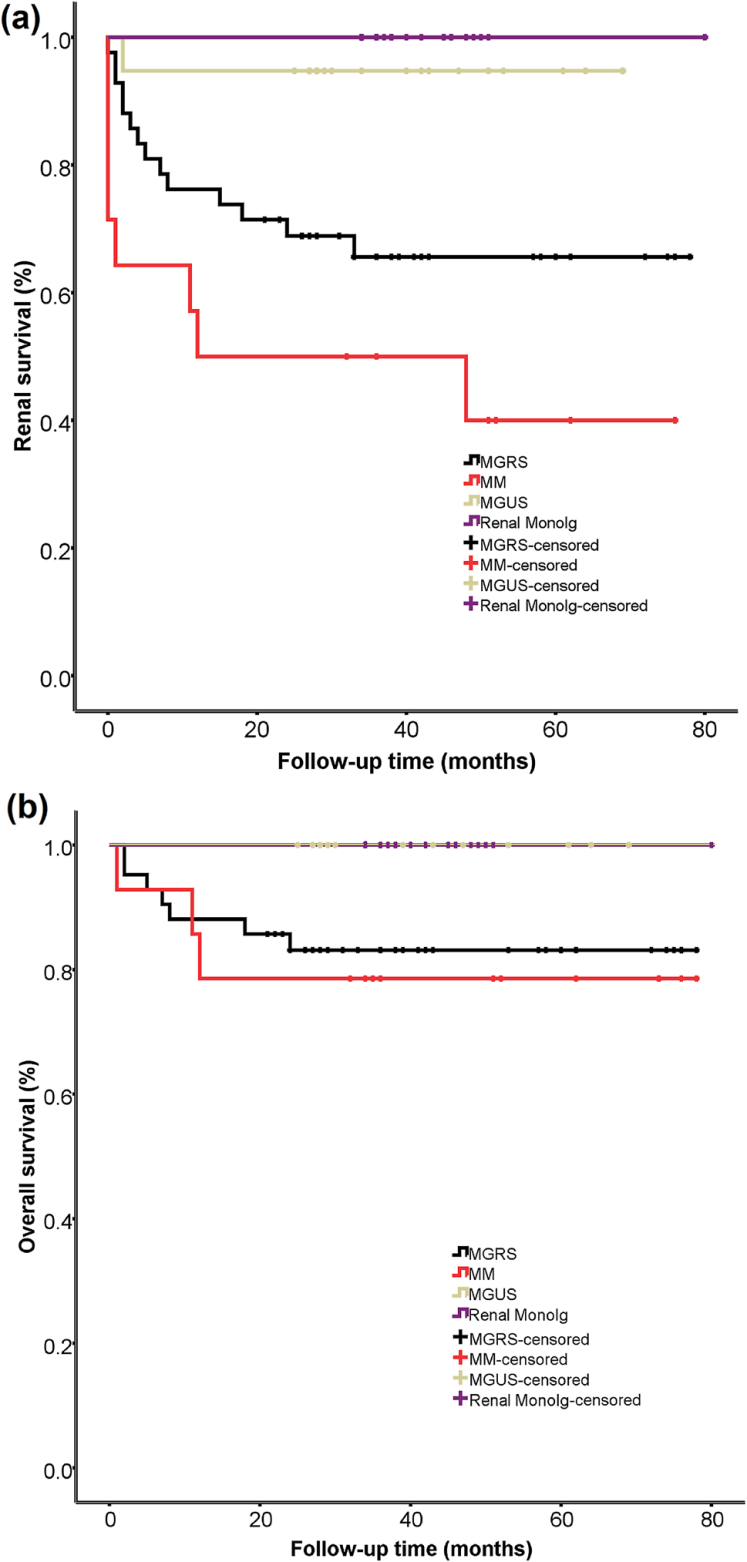
Comparison of renal survival **a** and overall survival **b** in patients with MGRS, MM, MGUS, and renal monoIg

**Fig. 4:**
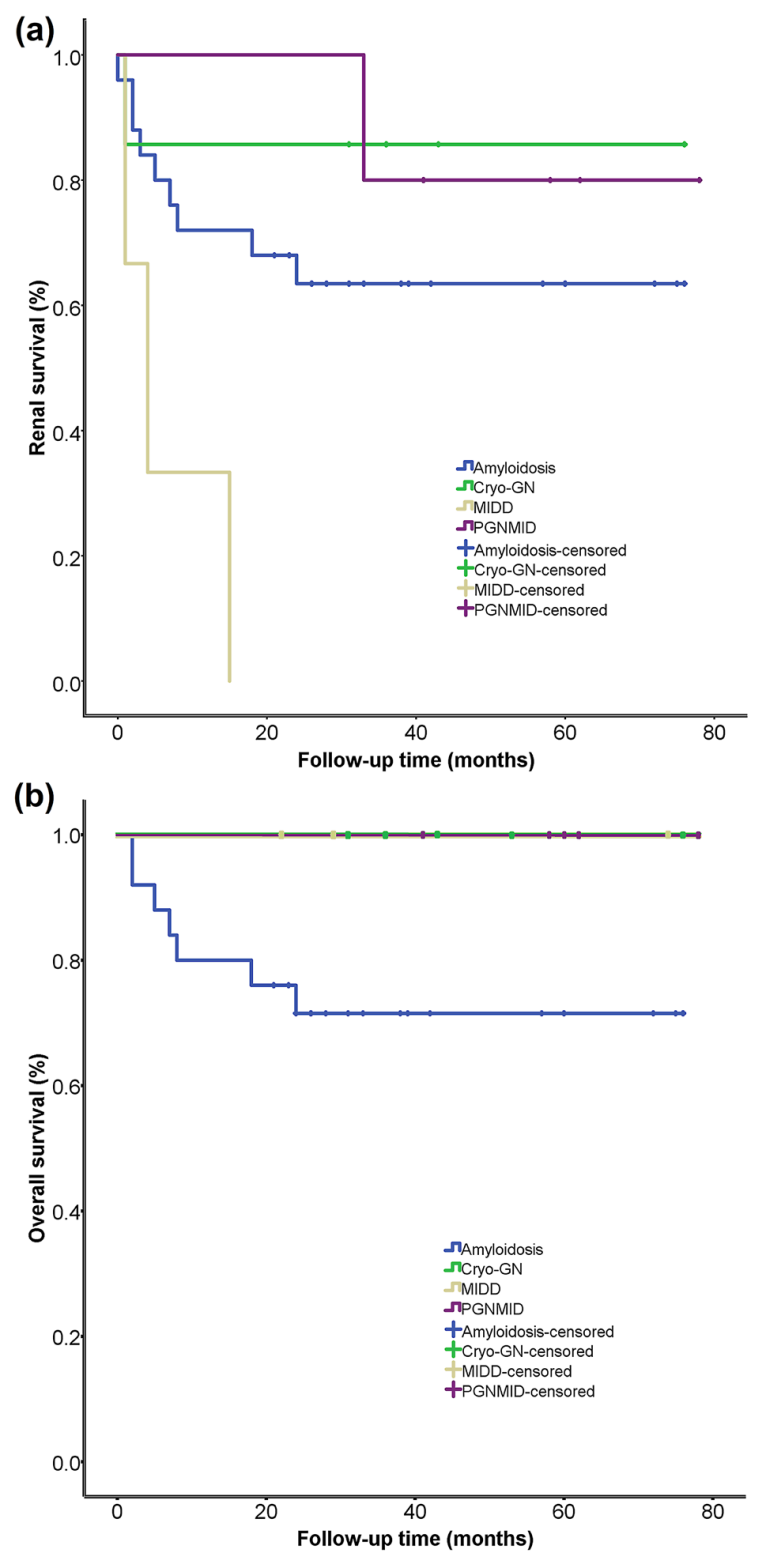
Comparison of renal survival **a** and overall survival **b** in patients with MGRS who have amyloidosis, Cryo-GN, MIDD, and PGNMID

By Kaplan–Meier analysis, renal survival was significantly better in the MGUS and renal monoIg groups than in the MGRS and MM groups (*P * <  0.05), although it had no significant difference between the MGRS and MM groups (*P * >  0.05; Fig. 3a). The overall survival was significantly better in the renal monoIg group than in either the MGRS or the MM group (*P * =  0.05 and *P * =  0.03, respectively), and it was also better in the MGUS group than the MM group (*P * =  0.04; Fig. 3b). Among the patients with MGRS, those with MIDD had much poorer renal survival compared with those in the other groups (*P * <  0.05), although the overall survival was not significantly different between them (Fig. 4).

Survival analysis identified eGFR, abnormal urine *κ*/*λ* ratio, and the presence of renal monoclonal light chains as risk factors, but they were not independent risk factors for mortality in the MGRS group. However, SCr, Hb, and the serum *κ*/*λ* ratio were identified as independent risk factors for ESRD in the MGRS group (Table 4). There were no risk factors for death in the MM group, and although SCr, eGFR, and interstitial fibrosis were risk factors, they were not independent for ESRD.

**Table 4:** Independent risk factors for ESRD of MGRS

	MGRS			
	HR	95% CI lower	95% CI upper	*P*
Scr	1.004	1.002	1.006	0.000
Hb	1.036	1.000	1.073	0.049
Serum *κ*/*λ* ratio	3.550	1.176	10.713	0.025

Univariate variables included the following variables: age, gender, lesion duration, hematuria, proteinuria, albumin, Hb, Scr, eGFR, low serum C3 level, abnormal serum and urine *κ*/*λ* ratio, abnormal sIFE and uIFE, presence of renal monotypic *κ*, glomerular global and segmental sclerosis, crescents, IFTA, interstitial inflammation, and podocyte effacement. Multivariate variables included Hb, Scr, eGFR, abnormal serum and urine *κ*/*λ* ratio, and glomerular global and segmental sclerosis

## Discussion

There is a higher prevalence of both MGUS and chronic kidney disease (CKD) in older patients. The same patient could therefore have unrelated hematological conditions and CKD. As such, distinguishing the conditions is essential because of their different clinical characteristics and associated therapies [17]. MGRS can only be confirmed by renal biopsy, which has been widely used to distinguish it from different types of renal injury. It has recently been reported that clinically suspected MGUS had 40% of patients progressing to MGRS, while another study showed only 10% years ago [6, 18]. In our study, 56%, 25.3% and 18.7% of patients with monoclonal gammopathy who underwent renal biopsy had MGRS, MGUS with unrelated CKD, and MM. Two (4.5%) patients who were initially diagnosed with MGRS progressed and were reclassified as having MM after a second bone marrow biopsy, although the progression rate was previously reported to be 18% [8]. Amyloidosis was diagnosed in 59.5% of patients with MGRS, which is consistent with the findings of previous studies [6, 19, 20] and may explain the lower albumin level and worse podocyte effacement in MGRS group than the other groups. Among the patients with MM, 64.3% had cast nephropathy, which is consistent with a previous study [21]. This likely explains the lower eGFR and Hb level, higher SCr level and paraprotein detection rate, and the worse chronic pathological lesions in the MM groups than in the other groups. In the present study, patients with MGUS were mainly diagnosed with primary IgAN, followed by IMN, which may explain the lower serum C3 levels and greater podocyte effacement in the MGUS group than in the other groups. Additionally, renal monoIg deposit diseases without hematological monoclonal gammopathy are poorly understood. Our study demonstrated that most (90.5%) of these patients had IgAN with *λ* restrictions, and they were much younger than patients with MGRS, MGUS, and MM. They had less proteinuria and lower Scr levels, and had more hematuria, but no paraprotein was detected in their serum or urine samples during follow-up. These data indicate mild pathogenicity in these patients compared to paraprotein-related kidney injuries. Boumediene et al. [22] compared monoclonal IgAN with primary IgAN and found that *λ* restricted IgA1 had hyposialylation, but no hypogalactosylation, which provides low isoelectric point (pI) and high affinity of mesangial region, causing selective deposition. However, Vignon et al. [10] reported that monotypic IgA deposits in such as heavy chain restricted IgAN and IgA-PGNMID exhibited distinct clinical, histological, and pathophysiological features from polytypic IgA deposits, with a 35.7% paraprotein detection rate according to IFE. Therefore, long-term follow-up is essential for patients with renal monoIg. Furthermore, there were two patients with LN and hepatitis B-related glomerulonephritis (HBV-GN), which showed *λ* restriction in renal tissue. We hypothesized that this was due to the selectivity difference of two light chains, according to the similar treatment and prognosis of LN or HBV-GN, and no MIg was detected during follow-up.

MGRS is complex and heterogenous, and manifests in diverse patterns of renal injury [13, 23, 24]. Consensus regarding the evaluation of MGRS was reached and released in 2019 [12]. In the present study, amyloidosis was the most common disease in patients with MGRS, followed by Cryo-GN, PGNMID, and MIDD. These findings were consistent with those of a previous study [20]. The incidence of ITG and C3GN is low in patients with MGRS [25, 26], and in this study we encountered only one case of each. Patients with amyloidosis had less hematuria, higher Hb levels, lesser proportion of abnormal *κ* chains, and milder IFTA and inflammation due to its fibril features without activation of the complement pathway [27]. Patients with Cryo-GN exhibited more proteinuria, lower eGFR, and a higher proportion of crescents than the others groups, which are indicators of nephritis [28]. Patients with MIDD had higher levels of albumin and SCr, more global sclerosis and interstitial fibrosis, and lower eGFRs, which might have led to worse prognosis [29]. Patients with PGNMID had the lowest paraprotein detection rate, which is consistent with the findings of a previous study [30], although the difference was not statistically significant. In the PGNMID group, SCr was the lowest and these patients had low serum C3 levels, which are indicators of complement pathway activation and better prognosis with immune suppressive treatment [31].

Most of our patients underwent special treatment including chemotherapy, and immunosuppressive therapy. Chemotherapy was more widely administered in the MM group than in the MGRS group, and patients with MM exhibited more partial renal response, but no major response. While SCr, Hb, serum *κ*/*λ* ratio were independent risk factors for ESRD in the MGRS group, no independent risk factors were found in the MM group. The overall mortality rate of MM and MGRS was low due to active chemotherapy and the clone-directed approach [32, 33]. In the MGRS group, immunosuppressive therapy was only administered in patients with Cryo-GN and PGNMID, because they had complement activated proliferative glomerulonephritis, and no detectable clone in serum was found in patients with PGNMID. These patients were empirically prescribed with immunosuppressive medications and were relatively responsive, similar to the findings of previous reports [34–37]. Patients with MGUS and renal monoIg only received immunosuppressive therapy and after 4 years of follow-up on average, most patients achieved a major response and none of them died. Burwick et al. [38] reported that the progression of renal malfunction in these patients with MGUS to ESRD was 27.6% with severely reduced eGFR versus 4.0% with preserved eGFR. However, only one (5.3%) patient progressed to ESRD in the MGUS group in our study, with slightly reduced eGFR. The overall survival was also reasonably high in the MGUS group, which was similar to that in the MGRS and MM groups, but renal survival was relatively low in the MM group. The Kaplan–Meier analysis revealed better renal and overall survival in the MGUS and renal monoIg groups than in the MGRS and MM groups, indicating that organ damage associated with MIg resulted in significantly worse outcomes. Additionally, patients with MIDD exhibited significantly poorer renal survival than patients with MGRS indicating that early diagnosis and aggressive therapy are especially important for these patients.

The limitations of this study were its retrospective design and the lack of long-term follow-up. Additionally, we were unable to analyze abnormal free light chain due to limited detection in a few patients, and complete hematological data could not be analyzed during follow-up.

## Conclusion

The clinicopathological changes in patients with MGRS were between those in patients with MM and MGUS. The treatment of both MGRS and MM was more intensive, and the overall mortality rate was low. Both MGUS and renal monoIg alone exhibited fewer clinicopathological characteristics than MGRS and MM, and the treatment was mostly focused on primary renal diseases.

## Data Availability

The datasets used or analyzed during the current study are available from the corresponding author on reasonable request.

## Abbreviations

MIg: Monoclonal immunoglobulin
MGUS: Monoclonal immunoglobulinemia of unknown significance
MGRS: Monoclonal gammopathy of renal significance
MM: Multiple myeloma
Renal monoIg: Monotypic immunoglobulin in renal tissue
IgAN: IgA nephropathy
IMN: Idiopathic membranous nephropathy
SLE: Systemic lupus erythematosus
IF: Immunofluorescence
Hb: Hemoglobin
SCr: Serum creatinine
sIFE/uIFE: Serum/urine immunofixation electrophoresis
IFTA: Interstitial fibrosis/tubular atrophy
ESRD: End-stage renal disease
SD: Standard deviation
ANOVA: Analysis of variance
LCDD: Light chain deposit disease
LN: Lupus nephritis
Cryo-GN: Cryoglobulinemic glomerulonephritis
PGNMID: Proliferative glomerulonephritis with monoclonal IgG deposits
MIDD: Monoclonal immunoglobulin deposition disease
C3GN: C3 glomerulonephritis
ITG: Immunotactoid glomerulonephritis
IS: Immunosuppression therapy
pI: Isoelectric point
HBV-GN: Hepatitis B-related glomerulonephritis
